# Toxicodynamic Assessment of Aqueous Neem (*Azadirachta indica* A. Juss) Seed Extract on Mortality and Carboxylesterase Activity in Key Organs of *Bombyx mori* L. Larvae

**DOI:** 10.3390/toxins17060304

**Published:** 2025-06-16

**Authors:** Ajin Rattanapan, Chuthep Phannasri, Chawiwan Phannasri, Patcharawan Sujayanont, Kattinat Sagulsawasdipan

**Affiliations:** 1 Department of Biology, Faculty of Science, Mahasarakham University, Kantharawichai District, Mahasarakham 44150, Thailand; 2 Center of Excellence for Mulberry and Silk, Mahasarakham University, Kantharawichai District, Mahasarakham 44150, Thailand; 3 Department of Preclinic, Faculty of Medicine, Mahasarakham University, Muang District, Mahasarakham 44000, Thailand; patcharawan.s@msu.ac.th; 4 Tropical Health Innovation Research Unit, Mahasarakham University, Muang District, Mahasarakham 44000, Thailand; 5 Department of Marine Science and Environment, Faculty of Science and Fisheries Technology, Rajamangala University of Technology Srivijaya, Trang 92150, Thailand; kattinat.s@rmutsv.ac.th

**Keywords:** botanical insecticide, non-target toxicity, organ-specific response, enzymatic activity, sericulture

## Abstract

Botanical insecticides derived from neem (*Azadirachta indica* A. Juss.) seeds have gained significant interest due to their sustainable characteristics and low environmental impact. However, their use in sericulture remains contentious due to the heightened sensitivity of domesticated silkworms to environmental stressors. This study systematically investigates the toxicodynamic effects of aqueous neem seed extract (ANSE) on fifth instar larvae of Thai multivoltine *Bombyx mori* L., focusing on larval mortality and carboxylesterase (CarE) enzyme activity in essential detoxification organs. Larvae were exposed to ANSE concentrations ranging from 5 to 50 mg L^−1^ for up to 72 h. Key findings highlight a pronounced dose- and time-dependent increase in mortality, with an accurately determined LC_50_ value of 17 mg L^−1^ at the longest time exposure, accompanied by mortality rates reaching approximately 83% at the highest concentration tested, indicating considerable susceptibility. Additionally, notable and distinct organ-specific responses were observed, with significant inhibition of CarE activity in the midgut contrasting with elevated activities in the fat body and Malpighian tubules. These differential enzymatic responses reveal previously undocumented adaptive detoxification mechanisms. Consequently, the study advocates cautious and regulated application of neem-based insecticides in sericulture, recommending precise management of concentrations and exposure durations according to silkworm strain sensitivities to ensure optimal silk production.

## 1. Introduction

In recent decades, botanical insecticides have increasingly attracted attention as sustainable alternatives to conventional synthetic pesticides, primarily due to their favorable environmental attributes, inherent biodegradability, and minimal toxicity towards non-target organisms [1,2]. Prominent botanical insecticides receiving global recognition include pyrethrins, derived from *Tanacetum cinerariifolium* (Trevir.); rotenone, extracted from species such as *Derris elliptica* (Wall.); and neem oil, derived from *Azadirachta indica* A. Juss. seed [3,4,5]. Among these, bioactive constituents from neem seeds (*A. indica*) have garnered particular interest owing to their diverse phytochemical profiles and extensive biological activities [6,7]. These compounds exert insecticidal effects primarily by disrupting vital physiological processes, ultimately causing insect mortality. Such disruptions typically involve complex mechanisms, including interference with essential enzymatic pathways, thus providing multifaceted modes of action against insect pests [8,9,10].

The ecological compatibility and effectiveness of neem-based insecticides within integrated pest management (IPM) strategies have facilitated their widespread agricultural adoption [11]. However, the use of neem-derived products in domesticated sericulture, *Bombyx mori* L., remains highly contentious. This controversy arises mainly from the heightened sensitivity of silkworm larvae to chemical agents, which are environmental stressors, potentially impairing their health and survival [12,13,14,15]. Previous research has documented significant negative impacts, particularly lethal effects, of neem exposure on silkworms and other Lepidopteran larvae, generating substantial economic concerns for silk production. These findings highlight the necessity of cautious neem-product application in sericulture [16,17,18,19]. Despite extensive studies, gaps remain in understanding the biochemical mechanisms and physiological responses underlying neem-induced toxicity, creating uncertainties regarding safe and sustainable neem utilization in sericulture.

A crucial aspect of insect responses to xenobiotics involves detoxification enzymes, serving as primary defense mechanisms against harmful substances [20]). Carboxylesterase (CarE), a major esterase group, plays a pivotal role in metabolizing and neutralizing various toxicants, including plant-derived toxins [21,22]. Enhanced CarE activity in resistant insect strains underscores its key defensive function [23,24]. In *B. mori*, CarE enzymes actively metabolize secondary metabolites from mulberry leaves, environmental contaminants, and dietary insecticides [25]. At least 76 CarE genes have been identified in *B. mori*, whic crucial for detoxifying dietary toxins and mitigating xenobiotic impacts. CarE enzymes exhibit maximal activity in primary detoxification organs, notably the midgut, fat body (analogous to vertebrate liver), and Malpighian tubules. Such strategic localization optimizes their detoxification efficiency by intercepting toxic compounds before they reach critical physiological targets, thus robustly protecting insects against xenobiotic toxicity [26,27]. Consequently, CarE serves as a reliable biomarker for assessing insect stress responses to chemical exposure, providing critical insights into adaptive biochemical mechanisms utilized against toxicants [28,29]. Additionally, given that silkworms exhibit toxicokinetic and toxicodynamic responses comparable to other animals, these studies possess broader biological implications [30].

Our previous research demonstrated significant inhibition of esterase (EST) activity by neem seed crude extract in the whole body of early instar larvae and specifically in the midgut of late instar larvae of hybrid polyvoltine silkworm strains, indicating adverse impacts on beneficial non-target organisms within sericulture. Moreover, fifth instar larvae exhibited greater tolerance compared to younger larvae. These results were accompanied by a concurrent increase in glutathione-S-transferase (GST) activity, suggesting compensatory biochemical responses [31]. The inhibition of detoxification enzymes, especially within critical detoxification organs such as the midgut, is of great interest in economically significant insects like silkworms due to the potential to cause substantial economic damage to sericulture farmers. Based on these findings and the aforementioned intriguing characteristics of CarE, the present study seeks to extend the investigation to indigenous polyvoltine silkworm strains, recognized for their higher environmental resilience compared to hybrid varieties [32]. Specifically, this research focuses on evaluating the effects of aqueous neem seed crude extract (ANSE) on mortality and carboxylesterase (CarE) enzyme activity in crucial detoxification organs—midgut, fat body, and Malpighian tubules—of fifth instar larvae. This developmental stage is characterized by rapid growth, significant physiological changes, nutrient accumulation, hormonal shifts, and preparation for pupation, making it an optimal model for elucidating functional impacts [33,34]. By systematically characterizing toxicodynamic interactions between neem-derived phytochemicals and silkworm physiological responses, this study aims to establish essential knowledge for developing safer and more effective pest management practices in sericulture, ultimately enhancing the sustainability and productivity of this economically significant industry.

## 2. Results

### 2.1. Effects of ANSE on Fifth Instar Silkworm Larvae

#### 2.1.1. Larvae Mortality

**Figure 1:**
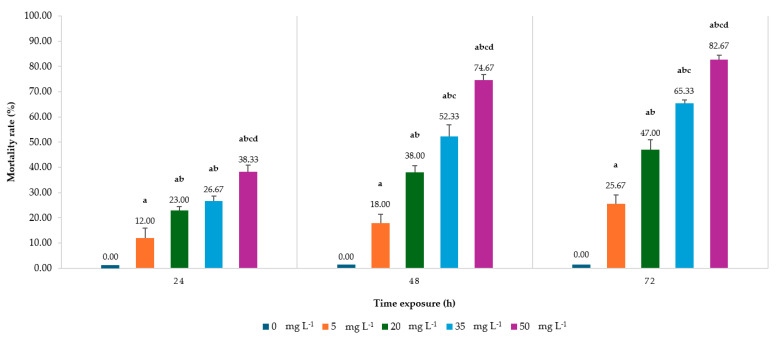
Mortality rate of fifth instar *Bombyx mori* larvae following exposure to ANSE at concentrations of 0, 5, 20, 35, and 50 mg L^−1^ for 24, 48, and 72 h is presented in the bar graphs. Data are expressed as mean ± standard deviation (SD). Statistical analysis was conducted using one-way ANOVA, followed by Tukey’s HSD post hoc test for multiple comparisons. Significant differences among treatment groups (*p* < 0.001) are indicated by the letters a, b, c, and d.

The acute toxicity of ANSE was evaluated on Thai multivoltine silkworm larvae across varying concentrations (5, 20, 35, and 50 mg L^−1^) and exposure durations (24, 48, and 72 h). Mortality rates demonstrated a clear concentration-dependent and time-dependent trend, with higher extract concentrations and longer exposure durations resulting in significantly increased larval mortality (*p* < 0.05). Figure 1 showed at 24 h post-exposure, the mortality rate was notably higher at higher concentrations of the extract. The treatment with 50 mg L^−1^ resulted in a mortality rate of 38.33 ± 2.64%, which was the highest recorded at this time point, followed by 35 and 20 mg L^−1^ with 26.67 ± 2.04 and 23.00 ± 1.39%, respectively. The lowest concentration, 5 mg L^−1^, caused a mortality rate of 12.00 ± 3.98%. These results indicate that even at the shortest exposure duration, the ANSE exhibited acute toxicity, with mortality increasing with concentration. By 48 h post-exposure, mortality rates had substantially increased. The extract at 50 mg L^−1^ led to a mortality rate of 74.67 ± 2.17%, nearly doubling the mortality observed at 24 h. Similarly, 35 mg L^−1^ caused 52.33 ± 4.50% mortality, and 20 mg L^−1^ resulted in 38.00 ± 2.74%. The mortality rate at 5 mg L^−1^ also increased to 18.00 ± 3.42%. The significant increase in mortality over time suggests a cumulative toxic effect of the extract, with its potency amplified as exposure duration extended. At 72 h post-exposure, the toxic effect of the extract was most pronounced. The highest concentration, 50 mg L^−1^, resulted in a mortality rate of 82.67 ± 1.90%, affecting over 80% of the larvae population. Similarly, 35 mg L^−1^ caused a mortality rate of 65.33 ± 1.39%, and 20 mg L^−1^ led to 47.00 ± 3.98%. Even the lowest concentration, 5 mg L^−1^, demonstrated a mortality rate of 25.67 ± 3.46%, highlighting the prolonged toxic effects of the extract. These results highlight the time-dependent toxicity of the extract, as mortality rates increased significantly with longer exposure durations. The control group (0 mg L^−1^) showed 0% mortality across all time points, confirming that the observed mortality in treated groups was solely due to the toxic effects of the ANSE. This provides a reliable baseline for comparison and highlights the extract’s significant impact on larval survival. These findings demonstrate the dose- and time-dependent acute toxicity of ANSE on silkworm larvae. Higher concentrations (35 and 50 mg L^−1^) and longer exposures showed stronger toxic effects on silkworm larvae.

#### 2.1.2. Survival Rate of Larvae

**Figure 2:**
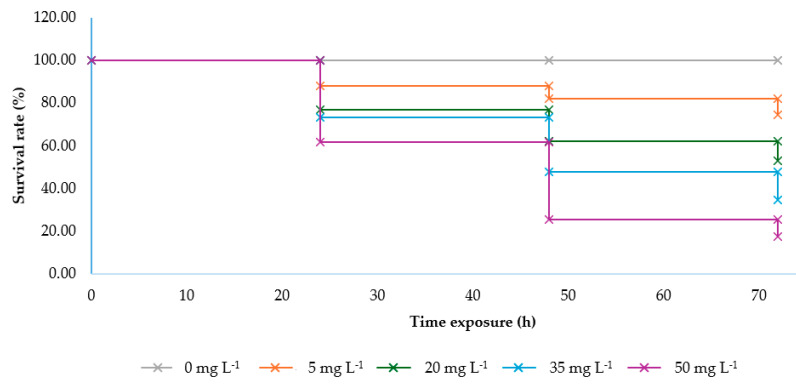
Survival rate (%) of fifth instar *Bombyx mori* larvae exposed to varying concentrations of ANSE over 72 h.

The survival rate of silkworms, as illustrated in Figure 2, varies depending on the concentration of ANSE and the duration of exposure, demonstrating a clear dose- and time-dependent pattern. The control group (0 mg L^−1^) consistently maintained 100% survival across all time points, confirming the absence of adverse effects without ANSE exposure. In contrast, the survival rate declined progressively with increasing concentrations and extended exposure times. At 5 mg L^−1^, the survival rate remained steady at 100% for the first 24 h but declined slightly to 88.00% by 48 h and further to 74.33% at 72 h, indicating mild toxicity. At 20 mg L^−1^, survival began to decrease after 24 h, dropping to 62.00% and 53.00% at 48 and 72 h, respectively, indicating moderate toxicity. At 35 mg L^−1^, the survival rate fell significantly, reaching 47.67% and 34.67% at 48 and 72 h, respectively, demonstrating severe toxic effects at this concentration. The most pronounced impact was observed at 50 mg L^−1^, where survival dropped sharply to 25.33% at 48 h and declined further to 17.33% at 72 h, reflecting the highest toxicity level. These findings highlight the potent toxic effects of ANSE, with higher concentrations and prolonged exposure resulting in greater reductions in silkworm survival. This underscores the importance of carefully controlling the concentration and exposure duration of neem-based products to minimize harm to silkworms.

#### 2.1.3. Toxicity Value

**Figure 3:**
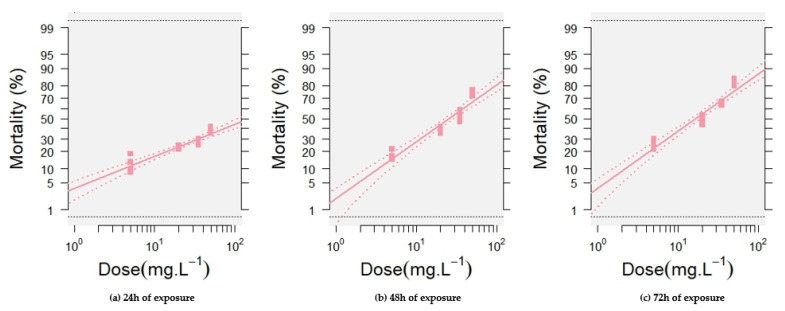
Linear regression analyses of probit-transformed mortality rates against the logarithmic concentrations of ANSE in fifth instar *Bombyx mori* larvae, with 95% confidence intervals. (**a**) Mortality following 24 h exposure; (**b**) mortality following 48 h exposure; and (**c**) mortality following 72 h exposure.

The probit analysis demonstrates a strong linear relationship between the log-transformed concentrations of ANSE and the mortality rates of silkworm larvae. At each exposure time (24, 48, and 72 h), the mortality increases with the concentration of the extract, and the regression lines exhibit a good fit, indicating the reliability of the model in predicting toxicity values. The slopes of these regression lines, presented in Figure 3 and Table 1, increased from 0.82 ± 0.12 at 24 h to 1.46 ± 0.13 at 48 h and then remained constant at 72 h, reflecting greater sensitivity of the larvae to the extract over longer durations. This trend confirms the cumulative toxic effect of ANSE on silkworm larvae as exposure time increases.

The LC_10_, LC_50_, and LC_90_ values in Table 1 further quantify the toxicity of the extract. After 24 h, the LC_50_ is 149 mg L^−1^, indicating that this concentration is required to cause 50% mortality within the initial period. The LC_10_ and LC_90_ for this time point are 4.11 and 5403 mg L^−1^, respectively, illustrating the wide range of concentrations affecting the larvae at this early time stage. By 48 h, the LC_50_ decreases significantly to 25 mg L^−1^, while the LC_10_ and LC_90_ also drop to 3.36 and 193 mg L^−1^, respectively. This substantial reduction highlights the increased lethality of the extract with prolonged exposure. After 72 h, the LC_50_ further declines to 17 mg L^−1^, while the LC_10_ and LC_90_ reach 2.20 and 125 mg L^−1^, respectively. These values demonstrate the heightened susceptibility of the larvae over time, with even lower concentrations causing significant mortality. These findings confirm that ANSE exhibits potent toxicity to *B. mori* larvae, with lethality intensifying over time and at higher concentrations. The dose–response relationship and probit analysis provide a robust framework for assessing the effects of the extract.

### 2.2. Effects of ANSE on CarE Activity in Fifth Instar Silkworm Larvae

#### 2.2.1. CarE Activity in Midgut

**Figure 4:**
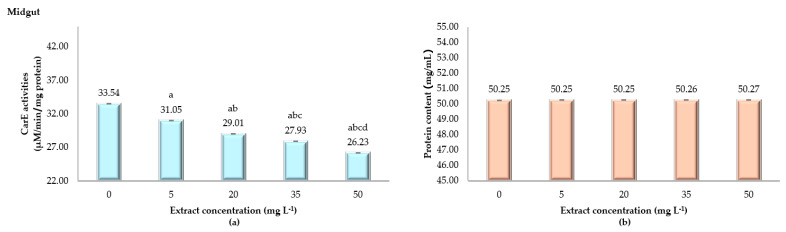
Carboxylesterase (CarE) activity (µM/min/mg protein) in the midgut (**a**) and protein content (**b**) of fifth instar *Bombyx mori* larvae following 24 h of exposure to varying concentrations of ANSE. The bar graphs display the means ± standard deviation (SD). Statistical differences among groups were assessed using one-way ANOVA, followed by Tukey’s HSD post hoc test for multiple comparisons. Significant differences among treatment groups (*p* < 0.001) are indicated by the letters a, b, c, and d.

The effects of ANSE on enzyme activity and protein content in the midgut of fifth instar silkworm larvae are highlighted in Figure 4. In the control group (0 mg L^−1^), enzyme activity is highest at 33.54 ± 0.02 µM/min/mg protein. Exposure to 5 mg L^−1^ reduces activity to 31.05 ± 0.01 µM/min/mg protein, with further decreases observed at 20, 35, and 50 mg L^−1^, showing 29.01 ± 0.01, 27.93 ± 0.02, and 26.23 ± 0.02 µM/min/mg protein, respectively. This dose-dependent reduction in enzyme activity indicates an inhibitory effect of the extract. Statistically, all treatment groups show significant reductions compared to the control (*p* < 0.001). Protein content, in contrast, remains consistent across all concentrations, with values ranging narrowly from 50.25 ± 0.01 to 50.27 ± 0.01 mg/mL. No statistical differences are observed among groups, suggesting the extract does not affect overall protein levels in the midgut. The data show a clear inverse relationship between extract concentration and enzyme activity, while protein content remains unaffected. This indicates that ANSE selectively inhibits enzyme activity in a dose-dependent manner without altering the protein content.

#### 2.2.2. CarE Activity in Fat Body

**Figure 5:**
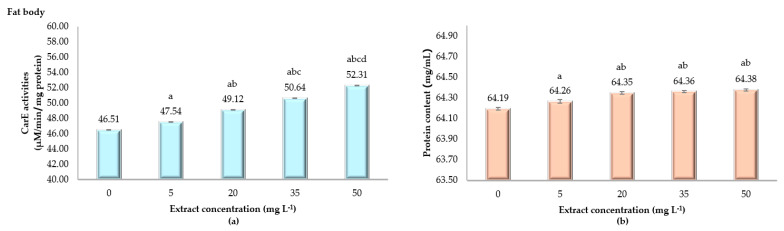
Carboxylesterase (CarE) activity (µM/min/mg protein) in the fat body (**a**) and protein content (**b**) of fifth instar *Bombyx mori* larvae following 24 h of exposure to varying concentrations of ANSE. The bar graphs display the means ± standard deviation (SD). Statistical differences among groups were assessed using one-way ANOVA, followed by Tukey’s HSD post hoc test for multiple comparisons. Significant differences among treatment groups (*p* < 0.001) are indicated by the letters a, b, c, and d.

The effects of ANSE on enzyme activity and protein content in the fat body of fifth instar silkworm larvae are highlighted in Figure 5. Enzyme activity in the control group (0 mg L^−1^) is 46.51 ± 0.01 µM/min/mg protein. Exposure to 5 mg L^−1^ slightly increases activity to 47.54 ± 0.01 µM/min/mg protein, with further dose-dependent increases observed at 20, 35, and 50 mg L^−1^, showing 49.12 ± 0.01, 50.64 ± 0.02, and 52.31 ± 0.01 µM/min/mg protein, respectively. Statistically, all extract concentrations show significant increases in enzyme activity compared to the control (*p* < 0.001). Protein content shows minimal increases across concentrations, with the control at 64.19 ± 0.01 mg/mL and slight elevations at 5, 20, 35, and 50 mg L^−1^ at 64.26 ± 0.02, 64.35 ± 0.01, 64.36 ± 0.01, and 64.38 ± 0.01 mg/mL, respectively). These differences are statistically significant (*p* < 0.001) but represent only minor changes. The data indicate a dose-dependent stimulatory effect of the extract on enzyme activity in the fat body, with protein content remaining relatively stable. This suggests a specific physiological response where enzyme activity increases while protein levels are largely unaffected.

#### 2.2.3. CarE Activity Malpighian Tubules

**Figure 6:**
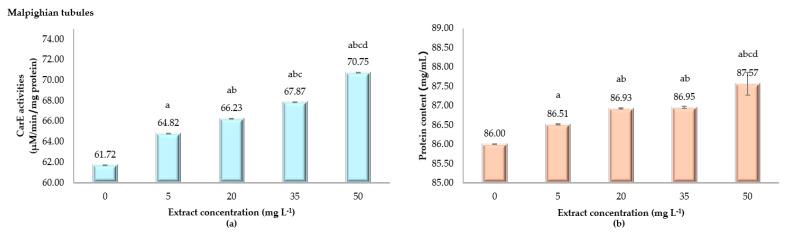
Carboxylesterase (CarE) activity (µM/min/mg protein) in the Malpighian tubules (**a**) and protein content (**b**) of fifth instar *Bombyx mori* larvae following 24 h of exposure to varying concentrations of ANSE. The bar graphs display the means ± standard deviation (SD). Statistical differences among groups were assessed using one-way ANOVA, followed by Tukey’s HSD post hoc test for multiple comparisons. Significant differences among treatment groups (*p* < 0.001) are indicated by the letters a, b, c, and d.

The effects of ANSE on enzyme activity and protein content in the Malpighian tubules of fifth instar silkworm larvae are shown in Figure 6. Enzyme activity in the control group (0 mg L^−1^) is 61.72 ± 0.01 µM/min/mg protein and increases in a dose-dependent manner to 64.82 ± 0.01, 66.23 ± 0.01, 67.87 ± 0.02, and 70.75 ± 0.01 µM/min/mg protein at 5, 20, 35, and 50 mg L^−1^, respectively. All treatment groups exhibit statistically significant increases compared to the control (*p* < 0.001). Protein content also rises with extract concentration, from 86.00 ± 0.01 mg/mL in the control group to 86.51 ± 0.01, 86.93 ± 0.02, 86.95 ± 0.03, and 87.57 ± 0.29 mg/mL at 5, 20, 35 and 50 mg L^−1^, respectively. Statistical differences are significant, particularly at higher concentrations (*p* < 0.001). Both enzyme activity and protein content show parallel, dose-dependent increases, suggesting a stimulatory effect of the ANSE on biochemical activity and protein synthesis in the Malpighian tubules. These findings highlight a clear physiological response to the extract.

## 3. Discussion

### 3.1. Effects of ANSE on Mortality and Toxicity in Silkworm Larvae

The mortality and survival data from this study exhibited clear dose- and time-dependent patterns, consistent with fundamental toxicological concepts including dose–response and cumulative toxicity [35,36]. These outcomes reinforce our prior findings [31], with both studies highlighting increased mortality at higher ANSE concentrations and prolonged exposure periods. However, the current research specifies the LC_50_ values more precisely, declining from 149 mg L^−1^ at the shortest period (24 h) to 17 mg L^−1^ at the longest time exposure (72 h), and demonstrates significant variability in larval susceptibility (LC_10_–LC_90_ range), complicating standardized management in sericulture. Previous studies corroborate these toxicological responses. Kumutha et al. [16] noted that substantial developmental impacts on *B. mori* from neem oil and Metacid exposure, while Singh et al. [18] observed adverse effects despite neem extracts accelerating larval mounting, underscoring the need for careful management practices [14,37]). Yu et al. [38] confirmed acute insecticide toxicity in silkworms, further supporting their known susceptibility. Azadirachtin, a key neem constituent, consistently induces significant physiological disruption. Silk gland development and altered spinning in *Spodoptera frugiperda* due to azadirachtin were impaired [39]. Similarly, Gaurav and Hassan [19] documented growth inhibition in *B. mori*, echoing their findings. Azadirachtin’s broad-spectrum toxicity was comprehensively reviewed, emphasizing its hormonal and physiological disruptions [8]. Supporting this, neem phytochemicals have demonstrated notable insecticidal effects of neem powders against adult bean weevil (*Acanthoscelides obtectus*), indicating broader efficacy [40].

Probit analysis in this study aligns methodologically with prior toxicological assessments [41,42], affirming the importance of precise LC_50_ measurements. Behavioral disruptions by neem limonoids reported in rice leaffolder (*Cnaphalocrocis medinalis*) further validate consistent neem bioactivity across species [43]. The sublethal effects of pesticides carry significant implications for sericulture economics. Xu et al. [44] observed biochemical and transcriptomic disruptions in *B. mori* after dinotefuran exposure, highlighting potential risks from neem applications. Developmental delays in silkworms exposed to neonicotinoids have been identified [45]. Additionally, azadirachtin-induced apoptosis in silkworm prothoracic glands has been reported, underscoring neem’s specific physiological impacts [17], complemented by midgut alterations observed by Roel et al. [46]. Thus, comprehensive evaluation of lethal and sublethal neem impacts remains crucial [47], particularly given the adaptability of hybrid silkworm strains [32]. Targeted studies addressing these detailed effects are necessary to refine neem usage strategies, optimize pest control efficacy, and ensure sustainable silk production.

However, the limited mortality response at the highest tested concentration (50 mg L^−1^) after 24 h, which did not exceed approximately 39%, significantly broadens the fiducial limits for LC_50_ and LC_90_ estimations, thus reducing the reliability of these derived lethal concentrations. This finding likely indicates a delayed onset of acute toxicity or suggests activation of detoxification processes within the larvae at shorter exposure durations. Therefore, interpretation of probit-estimated lethal concentrations for the 24-h period should be approached cautiously, emphasizing the importance of considering longer exposure times for more accurate toxicological assessments.

### 3.2. Effects of ANSE on CarE Activity in Essential Organs of Silkworm Larvae

CarE activity is a recognized biomarker for assessing adaptive biochemical responses or physiological disturbances resulting from xenobiotic exposure, reflecting organ-specific detoxification mechanisms in insects [48].

In the midgut, CarE enzymes play a crucial role in detoxifying ingested xenobiotics. The observed dose-dependent reduction in CarE activity, decreasing from 33.54 ± 0.02 to 26.23 ± 0.02 µM/min/mg protein at a concentration of 50 mg L^−1^, aligns closely with previous findings, indicating targeted disruption of detoxification pathways by neem seed extracts [31]. Similar decreases in enzyme activity have been reported in *B. mori* larvae exposed to phoxim [49] and dinotefuran [44], reflecting broader biochemical disruptions. Altered CarE gene expression associated with adaptive detoxification responses under viral stress (BmDNV-Z) was documented in *B. mori* by Gao et al. [24]. Neem-derived compounds consistently inhibit midgut CarE enzymes across various Lepidoptera. Significant reductions in midgut esterase activity were observed in *Glyphodes pyloalis* exposed to neem pesticides [50], and substantial CarE suppression was documented in *S. litura* following azadirachtin exposure [51]. Additionally, midgut structural and enzymatic disruptions in *S. frugiperda* larvae were treated with neem oil. [46]. Cytotoxicity and enzyme inhibition were also confirmed in *Anticarsia gemmatalis* larvae [52], while midgut esterase inhibition in *S. litura* exposed to *Melia toosendan* extracts, a neem-related plant [53], underscores neem’s broad enzyme-inhibitory potential. Genomic studies further support these biochemical findings, demonstrating differential CarE gene expression in *B. mori* and suggesting genetic regulation of detoxification responses [25,28]. Similarly, CarE gene disruption has been linked to reduced detoxification capacity in *Dendroctonus armandi* [54]. Despite strong enzyme inhibition observed in our study, stable protein levels suggest specific enzyme–substrate interference rather than broad protein synthesis disruption, aligning with targeted detoxification regulation via pathways such as reactive oxygen ROS/CncC described by Zeng et al. [55]. Collectively, these findings confirm neem extracts’ targeted insecticidal efficacy through midgut detoxification enzyme inhibition, highlighting their potential in pest management while underscoring the importance of cautious application to protect beneficial insects such as silkworms.

Conversely, the fat body exhibited a significant dose-dependent increase in CarE activity following ANSE exposure, rising from 46.51 ± 0.01 to 52.31 ± 0.01 µM/min/mg protein. This elevation represents an adaptive physiological response consistent with the established role of the fat body in metabolic regulation and detoxification [56,57]. This result aligns with the elevated detoxification enzyme activities in the fat body of *B. mori* following chlorantraniliprole exposure [58], indicative of systemic metabolic adjustments to xenobiotic stress. Similarly, the CarE activity significantly increased in the fat body of *B. mori* exposed to dinotefuran [44], highlighting this tissue’s metabolic responsiveness and sensitivity to toxicants. Further support is provided by Zhang et al. [59] and Wang et al. [49], who documented analogous elevations in fat-body CarE activities in silkworm larvae exposed to quercetin and phoxim, respectively, reinforcing the enzyme’s responsiveness to diverse chemical stressors. Additional evidence demonstrated shifts in glycogen metabolism within the fat body of *B. mori* exposed to organophosphorus insecticides, suggesting that prolonged enzymatic induction may incur metabolic trade-offs affecting other essential physiological processes [60]. Comparable enzymatic responses have been documented in other Lepidoptera; for instance, the elevation of CarE activity in the fat body of *S. litura* larvae following exposure to soybean-derived inhibitors [61]. Genomic studies further elucidate these biochemical adaptations; Tsubota and Shiotsuki [28] described differential expression of CarE genes in *B. mori* under xenobiotic exposure, and Birner-Gruenberger et al. [62] showed expression of multiple CarE genes in the fat body of *Drosophila melanogaster*, indicating their critical role in detoxification. Additionally, numerous CarE and related proteins in *Manduca sexta* under chemical stress were identified, highlighting a conserved genetic basis across Lepidoptera [63]. Nevertheless, prolonged enzyme induction may impose significant energetic costs, diverting metabolic resources away from critical processes such as growth, ultimately reducing overall insect fitness [56,57]. Therefore, further research into the chronic physiological effects of sustained detoxification enzyme induction is essential for ensuring the sustainable and effective integration of neem-based biopesticides into sericultural management practices.

In the Malpighian tubules, the observed dose-dependent increase in CarE activity, from 61.72 ± 0.01 to 70.75 ± 0.01 µM/min/mg protein at the highest ANSE concentration, underscores their critical role in detoxification and excretion processes [26]. This finding aligns with reports of elevated detoxification enzyme activities in the Malpighian tubules of *B. mori* larvae fed artificial diets, demonstrating their adaptive responses to dietary xenobiotics [64]. Similarly, structural and functional modifications of insect Malpighian tubules exposed to pesticides further confirm their importance in mitigating toxic stress [65]. The structural and physiological diversity of Malpighian tubules across insect species enables efficient xenobiotic excretion, thus protecting internal tissues [66]. Genomic evidence also indicates that Malpighian tubules in insects like *D. melanogaster* actively regulate detoxification enzyme expression under chemical stress, facilitating efficient toxin removal [67]. Direct interactions between detoxification mechanisms and excretory functions in Malpighian tubules of *D. melanogaster* show enhanced toxin excretion associated with elevated detoxification enzyme activities [68]. At the biochemical level, Wheelock and Nakagawa [48] described CarE enzymes as crucial components in xenobiotic metabolism, noting their broad adaptability across diverse toxicants, consistent with the increased CarE activity observed in this study. Furthermore, proteomic analyses revealed increased expression of multiple detoxification-related proteins in the Malpighian tubules of *B. mori* larvae under environmental stress, validating these organs’ central role in detoxification pathways [69]. Our study similarly recorded significant elevations in protein expression. Nevertheless, prolonged enzymatic induction may entail substantial metabolic costs, potentially impairing larval growth, developmental timing, and cocoon quality. Therefore, further investigations into the long-term physiological effects of sustained enzyme upregulation are necessary to facilitate the safe and sustainable integration of neem-derived biopesticides in sericultural practices.

Overall, the findings demonstrate that ANSE induces dose- and time-dependent toxicity, significantly altering CarE activity in key detoxification organs: midgut, fat body, and Malpighian tubules of fifth instar *B. mori* larvae. Accurate LC_50_ values revealed substantial variability in larval susceptibility, complicating standardized sericulture management. Organ-specific changes in CarE activity were evident: decreased midgut activity indicated disrupted detoxification pathways, while increased activity in the fat body and Malpighian tubules suggested systemic compensatory responses. A notable limitation of the study is its short-term scope, potentially overlooking chronic impacts. Genetic variability among silkworm strains significantly influences enzymatic responses, as confirmed by comparisons with our previous research. This finding underscores the importance of genotype-specific management strategies to optimize sericulture practices. Economically, the adverse effects of ANSE highlight the necessity for cautious application of neem-based biopesticides in sericulture. Excessive enzyme induction may negatively impact larval growth and cocoon quality, emphasizing precise dosage management to enhance sustainability and silk production efficiency.

## 4. Conclusions

This study determined that ANSE caused significant dose- and time-dependent toxicity in fifth instar *B. mori* larvae, clearly demonstrated through accurately quantified LC_50_ values. A novel aspect of this study is the distinct organ-specific responses in CarE enzyme activity: a notable inhibitory effect in the midgut, indicating impaired detoxification pathways, alongside increased enzyme activities in both the fat body and Malpighian tubules, suggesting compensatory physiological adaptations. These findings provide valuable and novel insights into the toxicodynamics of ANSE on Thai multivoltine silkworm strains, highlighting the need for cautious management practices in sericulture. Further investigation into long-term exposure effects and genotype-related differences could enhance understanding and guide safer application of neem-based insecticides in silk production.

## 5. Materials and Methods

### 5.1. Silkworm Rearing

Thai multivoltine silkworm (*B. mori*), a purebred strain, was evaluated in this study. Silkworm eggs were procured from the Queen Sirikit Sericulture Center in Roe-et Province, Thailand. The larvae were subsequently reared at the Silk Innovation Center, Mahasarakham University, Thailand. Throughout the experimental period, environmental conditions were carefully maintained at an optimal temperature of 25–27 °C, relative humidity of 70–80%, and a 12:12 h light–dark photoperiod. Fresh, healthy, pesticide-free mulberry leaves (*Morus alba* L.) were used as feed, provided three times daily. The rearing trays were lined with paraffin paper to ensure hygiene, and procedures for bed cleaning, spacing, and feeding were meticulously followed based on the modified methods outlined in our previous study [31]. The study utilized fifth instar larvae, selecting uniform, two-day-old, and three-day-old specimens for the assessment of toxicity and carboxylesterase activity, respectively.

### 5.2. ANSE Preparation

A specimen of *Azadirachta indica* A. Juss. was collected from the same location, and the extraction process was conducted following the methodology described in our previous research [31]. Briefly, raw seeds were thoroughly cleaned, and the seed coats were separated from the kernels. The seeds were then dried in a hot air oven (Memmert-600) at 60 °C for 48 h and subsequently ground into a fine powder using an electric grinder (Mxbaoheng Instrument Company, China). The neem seed powder was extracted through a maceration process using distilled water at a 1:4 ratio for one week at room temperature (36–37 °C), following a modified protocol adapted from Tabassam et al. [70]. The resulting aqueous extract was filtered using Whatman filter paper No. 1 and concentrated with a rotary evaporator. The crude extract was freeze-dried and stored at 4 °C until further experimentation.

### 5.3. Toxicity Assessment

Aqueous extracts were prepared at four concentrations: 5, 20, 35, and 50 mg L^−1^ for the bioassay. The concentration range used in the experiment was determined based on preliminary experiments following the methodological framework recommended by Finney [71] and WHO [72] to identify suitable exposure levels (unpublished data). The study aimed to evaluate the toxic effects of neem seed crude extract on fifth instar silkworm larvae. Toxicity assessment was conducted using a completely randomized design with five replicates, each consisting of 60 silkworm larvae. The assay employed a leaf-dipping method, modified from Rattanapan and Sujayanont [31] and Chen et al. [45]. The experiment was carried out in the laboratory of the Biology Department, Faculty of Science, Mahasarakham University, Thailand, under laboratory conditions consistent with those established for insect rearing. Fresh and healthy mulberry leaves were cleaned with distilled water, air-dried, and cut into 3 cm diameter discs. Each disc was immersed in a specific concentration of the plant extract solution for one minute and air-dried at room temperature for five minutes before being fed to the larvae. Control group leaves were treated with distilled water only. Larvae were housed individually in plastic boxes (one per box) and fed with the treated leaf discs. Following each replicate, the larvae were reared together in silkworm-rearing baskets. Treated mulberry leaf discs were provided to the silkworms during the morning feeding on the second day of the instar, followed by untreated and non-toxic foliage for subsequent feedings. The control group silkworms were fed mulberry leaves treated only with distilled water. Mortality was recorded at 24, 48, and 72 h post-exposure to assess toxicity. The exposure durations for acute toxicity assessment followed standard toxicological guidelines, typically involving short-term intervals (24–96 h) to accurately determine immediate toxic responses, such as LC_50_ [35]. Larvae were classified as dead if they exhibited no movement when probed with a brush. Data analysis was conducted using standard probit analysis, as described by Finney [71].

### 5.4. CarE Activity Evaluation

#### 5.4.1. Extraction of CarE

(1) Silkworm Tissue Preparation

Three-day-old fifth instar larvae were used for enzyme activity determination, as this is the most active developmental stage for silkworms. Three tissues, namely the midgut, fat body, and Malpighian tubules were investigated. To prepare silkworm tissues, the larvae were first immobilized using gentle restraints to ensure tissue integrity. Under a dissecting microscope, sterilized fine-grade scissors and forceps were used to make a longitudinal incision along the dorsal midline. The cuticle was retracted to expose the internal anatomy. Tissue dissection was performed following a modified method based on Rattanapan and Sujayanont [31], Wang et al. [49], Kaneko et al., and Wu et al. [73,74]. For midgut dissection, four midguts were collected per sample (0.12 g tissue per insect). The midgut, identifiable by its tubular structure, was carefully excised using sterilized forceps, avoiding disruption to the surrounding tissues. Each tissue was rinsed in 5 mL of pre-chilled phosphate-buffered saline (PBS, pH 7.5) to remove residual food and debris. For the fat body, similar dissection steps were followed to expose the body cavity. The fat body, appearing as a lipid-rich white structure along the body wall, was carefully excised using sterilized forceps. The excised tissue was rinsed in 5 mL of ice-cold PBS (pH 7.5) containing 1 mM PMSF (phenylmethylsulfonyl fluoride) to inhibit protease activity. The Malpighian tubules, located at the gut junction, were identified as fine, thread-like structures. Using fine-point forceps, the tubules were teased out under a dissecting microscope. They were then rinsed in 2 mL of PBS (pH 7.5) containing 1 mM PMSF.

(2) Enzyme Extraction

The extraction process followed the prescribed biological testing protocols for both treated and control groups, as outlined by Rattanapan and Sujayanont [31] and Yooboon et al. [75]. After a 24 h exposure period for each extract concentration, surviving larvae were used for enzyme extraction to assess enzyme activities. For each tissue, the sample was weighed and homogenized in a chilled mortar and pestle at a ratio of 1:10 (*w*/*v*). Homogenization was performed five times for 30 s each, with 30-s intervals, on ice, using 1 mL of 0.1 M potassium phosphate buffer containing 1 mM EDTA (pH 7.5) to ensure thorough disruption of cellular structures. The homogenate was then centrifuged at 10,000× *g* for 5 min at 4 °C. The supernatant containing the enzyme was carefully collected and transferred to a pre-chilled microtube. The samples were kept on ice for immediate investigation of carboxylesterase enzyme activity.

#### 5.4.2. Assay of CarE Activity Elucidation

Enzyme assays were conducted in vivo. CarE activity was measured using a modified p-nitrophenyl acetate (pNPA) assay, as described by Rattanapan and Sujayanont [31] and Paul et al. [76]. To prepare the enzymatic assay, para-nitrophenyl acetate (pNPA) was dissolved in anhydrous acetonitrile to create a 10 mM stock solution. For the working solution, 100 μL of the stock solution was diluted in 900 μL of 50 mM Tris-phosphate buffer (pH 7.5) to achieve a final concentration of 1 mM. In a quartz cuvette, 50 μL of enzyme extract, 850 μL of phosphate buffer (pH 7.5), and 100 μL of the 1 mM pNPA substrate were combined and mixed gently to ensure uniform distribution. The final reaction volume was 1 mL. The cuvette was placed in a spectrophotometer (Thermo Fisher Scientific, China) and pre-equilibrated to 30 °C. Enzyme activity was measured in kinetic mode by recording the absorbance at 410 nm for 60 s. The release of p-nitrophenol, indicated by the increase in absorbance, was used to calculate enzymatic activity. The molar extinction coefficient of p-nitrophenol (ε = 17,000 M^−1^ cm^−1^) was applied for this calculation. Control samples did not include enzyme extract.

#### 5.4.3. Protein Content Determination

The total protein levels were determined by the Bradford method [77], with bovine serum albumin as a standard. Measurements were recorded at 595 nm absorbance.

### 5.5. Data Analysis

All variables were expressed as means with standard deviations (SD). Mortality rates of fifth instar silkworm larvae exposed to different extract concentrations at 24, 48, and 72 h were analyzed using one-way analysis of variance (ANOVA), followed by Tukey’s post hoc tests for pairwise comparisons. Probit regression analysis was conducted using the BioRssay package [78] to calculate lethal concentrations (LC_10_, LC_50_, and LC_90_) along with their 95% confidence intervals (CI). Variations in carboxylesterase (CarE) activity and protein content in the midgut, fat body, and Malpighian tubules were analyzed using one-way ANOVA, followed by Tukey’s HSD post hoc analysis. Statistical significance was set at *p* < 0.001, with significant differences between groups indicated by the letters a, b, c, and d in the results. All statistical analyses were performed using R software (version 4.1.0).

## Figures and Tables

**Table 1:** Lethal concentrations (LC_10_, LC_50_, and LC_90_) of ANSE (mg L^−1^) against fifth instars of *Bombyx mori* larvae following a 72 h treatment period.

Time Exposure (h)	Slope ± SE	Intercept ± SE	Toxicity Value of ANSE (mg L^−1^)		
			LC_10_ (CI)	LC_50_ (CI)	LC_90_ (CI)
24	0.82 ± 0.12	(−1.79) ± 0.16	4.11 (1.50–24.00)	149.00 (25.00–3370.00)	5403.00 (423.00–476,627.00)
48	1.46 ± 0.13	(−2.05) ± 0.18	3.36 (1.69–9.06)	25.00 (9.37–108.00)	193.00 (52.00–1296.00)
72	1.46 ± 0.12	(−1.78) ± 0.16	2.20 (1.25–4.82)	17.00 (7.09–54.00)	125.00 (40.00–610.00)

The mortality rates of fifth instar *Bombyx mori* larvae exposed to different concentrations of ANSE were evaluated using probit analysis at 24, 48, and 72 h. Lethal concentrations in terms of LC_10_, LC_50_, and LC_90_ were determined and reported along with their corresponding 95% confidence intervals.

## Data Availability

The original contributions presented in this study are included in this article. Further inquiries can be directed to the corresponding author.

## Abbreviations

The following abbreviations are used in this manuscript:

ANSE	Aqueous neem seed extract
h	Hour
